# Complete mitochondrial genome sequence of the South Korean Eurasian eagle-owl, *Bubo bubo spp. kiautschenis* (Strigiformes; Strigidae)

**DOI:** 10.1080/23802359.2025.2528345

**Published:** 2025-11-09

**Authors:** Kwang-Bae Yoon, Yeong-Deok Han, Dong-Hyuk Jung, Ki-Yoon Kim, Yung-Chul Park

**Affiliations:** ^a^ Research Center for Endangered Species, National Institute of Ecology, Yeongyang-gun, Republic of Korea; ^b^ College of Veterinary Medicine, Chungbuk National University, Cheongju-si, Republic of Korea; ^c^ Division of Forest Science, College of Forest & Environmental Sciences, Kangwon National University, Chuncheon, Republic of Korea

**Keywords:** Mitogenome, *Bubo bubo*, phylogenetic analysis, Strigidae

## Abstract

Genomic data of the Eurasian eagle-owl (*Bubo bubo Kiautschenis; B. bubo*), an endangered species in South Korea is lacking. This study presents its first complete mitochondrial genome of *Bubo bubo* (GenBank accession no. OR756278). The resulting mitochondrial genome was 18,957 bp long and included two ribosomal RNAs, 13 protein-coding genes, 23 transfer RNAs, and two control regions, respectively. The mitogenome of *B. bubo* consisted of 29.6% A, 22.5% T, 33.8% C, 14.1% G, and 52.1% AT. Phylogenetic analysis revealed that *B. bubo* individuals were well grouped within the Strigidae.

## Introduction

**Figure 1.**
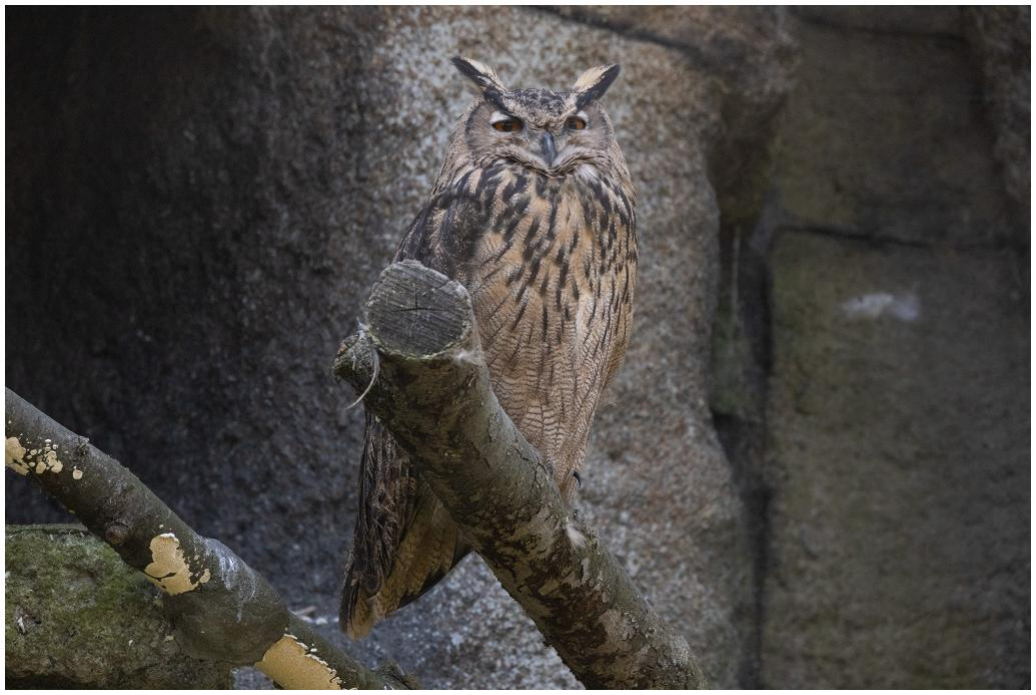
Specimen image of the Eurasian eagle-owl *Bubo bubo.* The image was provided by the National Institute of Ecology (NIE).

The Eurasian eagle-owl, *Bubo bubo* (*B. bubo spp. Kiautschenis*; Linnaeus, 1758) is among the largest owl species in the Strigidae family (Figure 1). It is distributed across East Asia, including South Korea and China, to Southern Russia, Central Asia, and Europe (Andrews 1990; Hengjiu et al. 2016; Meng et al. 2020). This bird inhabits rocky mountains, cliffs, forests, agricultural areas, urban environments, etc. (Dalbeck and Heg 2006; Smirnov and Kropacheva 2019). In South Korea, the Ministry of Environment has classified *B. bubo* as a Grade II endangered species, considering it a national cultural heritage (Kim et al. 2012). This designation underscores the criticality of protecting and conserving this species owing to its declining population and the increasing threats to its habitats (National Biodiversity Center 2019; Cultural Heritage Administration 2022). Despite being subjected to imminent extinction, the genomic data of this bird are unavailable. Thus, in this paper, we report the complete mitochondrial genome (mitogenome) of South Korean *B. bubo*, thus offering a reference for studies on the phylogenetic and evolutionary characteristics of this group.

## Materials and methods

The blood samples employed in this study were obtained from rescued Eurasian eagle-owls in Chungju-si, Chungcheongbuk-do, South Korea (38°07′18.4 N 128°10′10.2 E). These samples were deposited at the Wildlife Center of Chungbuk of the Chungbuk National University, Chungbuk-do, under voucher number CNUBB-22-01 (sample collector and administrator: Prof. Dong-Hyuk Jung/africabear@cbnu.ac.kr).

For the analysis, anticoagulated blood (10 µL) was added to the DNeasy Blood & Tissue Kit (Qiagen Inc., Hilden, Germany), after which genomic DNA (gDNA) was extracted using the manufacturer’s protocol. Further, the extracted gDNA was quantified using the NanoDrop One Microvolume UV–Vis Spectrophotometer (Thermo Fisher Scientific Inc., Wilmington, DE).

Thereafter, 28 complete mitogenome sequences (Harrison et al. 2004; Mahmood et al. 2014; Sun et al. 2016; Zhang et al. 2016; Kang et al. 2018; Lee et al. 2018; Liu et al. 2019; Park et al. 2019; Margaryan, unpublished data) belonging to the Strigidae family were retrieved from the GenBank database of the National Center for Biotechnology Information (https://www.ncbi.nlm.nih.gov/) and aligned using ClustalW in BioEdit v.7.2 (Hall 2011). Next, the raw FASTQ files were analyzed using FastQC v0.12.1 (Andrews 2010), after which they were imported into Geneious Prime v.2023.2.1 (Kearse et al. 2012) for quality control and assembly. The raw reads were preprocessed into Geneious Prime by merging paired ends, removing duplicates, discarding reads shorter than 50 bp, and trimming low-quality ends using the BBDuk trimmer by default from the BBTools plugin (Bushnell 2014). Afterward, the trimmed reads were assembled via a ‘map-to-reference’ approach using a validated *B. bubo* mitogenome available in the GenBank database (Accession No. MG681083) in the default setting.

Thereafter, the assembled consensus sequences obtained from the variant-calling process were visualized and annotated using Geneious Prime. The exact start-and-stop codons of all the protein-coding genes (PCGs) as well as the boundary of two ribosomal RNA (rRNA) genes were identified after aligning the newly analyzed complete mitochondrial sequence (in this study) with 19 other sequences (belonging to the Strigidae family retrieved from the GenBank database) using ClustalW multiple alignments in BioEdit v.7.2.

Additionally, maximum-likelihood (ML) analysis was performed using RAxML 7.0.4 (Stamatakis 2006; Stamatakis et al. 2008). A concatenated nucleotide matrix comprising all the PCGs (excluding the ND6 gene) was used to construct the phylogenetic tree. Employing a single program run, we explored the best-scoring ML tree (the ‘‐fa’ option) via an RAxML search. Finally, the best-scoring ML tree from thorough ML analysis was determined using the GTRGAMMAI model using 200 inferences. Statistical support was evaluated using 1000 nonparametric bootstrap inferences. FigTree 1.4.4 shows the resulting tree (Rambaut 2010).

## Results

**Figure 2.**
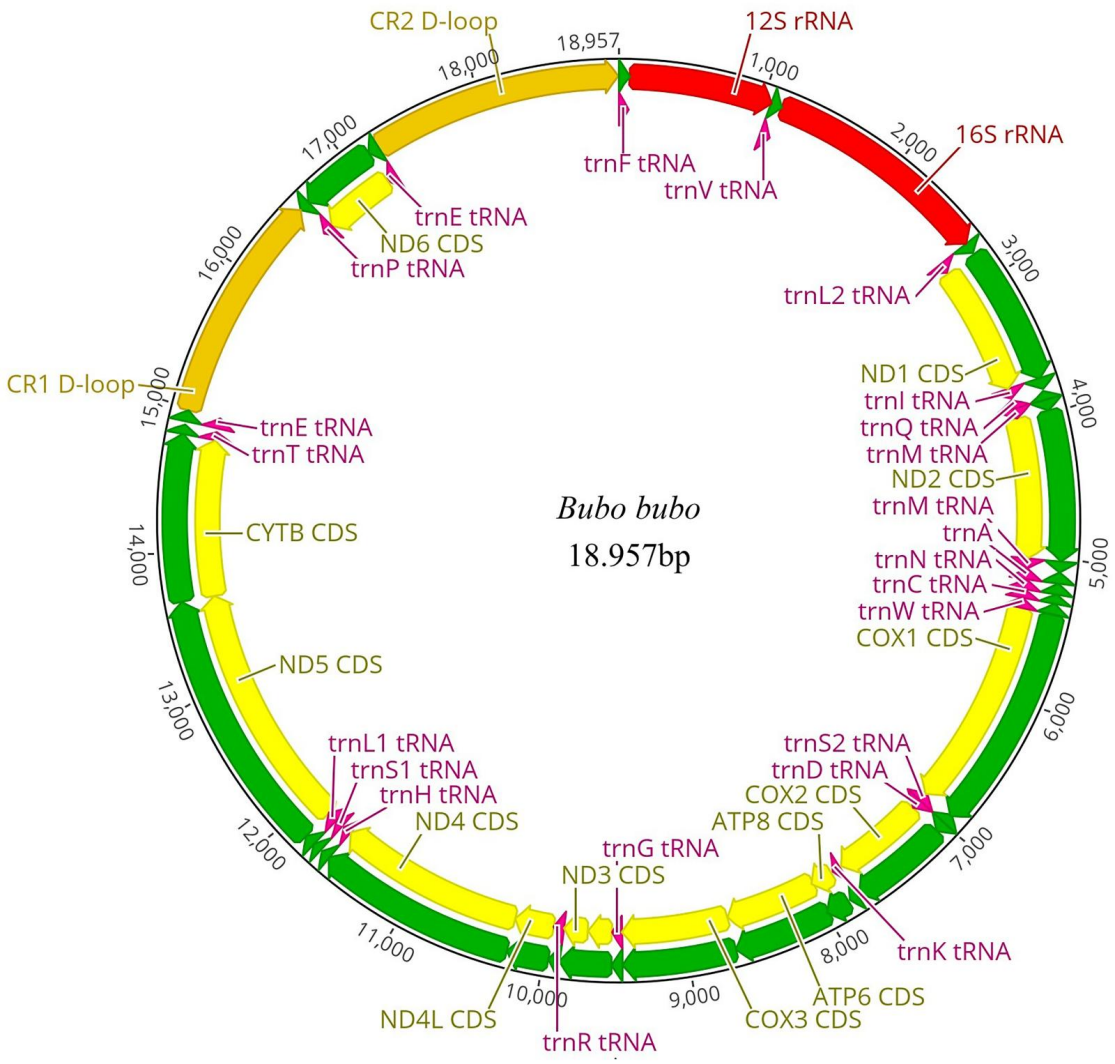
The mitochondrial genome of the Eurasian eagle-owl *Bubo bubo* (GenBank Accession No. OR756278). The 13 protein-coding genes, 23 tRNA, and two rRNA genes are shown in yellow, pink, and red, respectively.

As shown in Figure 2, the complete mitogenome of *B. bubo* (GenBank Accession No. OR756278) was 18,957 bp long. It comprised two rRNAs (*12S* and *16S rRNAs*), 13 PCGs, 23 transfer RNAs (tRNAs), and two control regions, CRs (CR1 and CR2).

The base composition of the mitogenome sequence was AT-biased, with nucleotide compositions of 29.6% A, 22.5% T, 33.8% C, 14.1% G, and 52.1% AT. Further, 12S and 16S rRNAs were 971 bp (52.1% A + T) and 1602 bp (53.4% A + T) long, respectively.

Further, the total length of the 13 mitochondrial PCGs of *B. bubo* was 14,187 bp (57.2% A + T), encoding 3797 amino acids without stop codons. All the PCGs were initiated with ATG except ND3 (ATA), ND5 (ATA), and ND6 (CAT). Furthermore, 23 tRNA genes, including two leucine-tRNA genes (*tRNALeu (CUN)* and *tRNALeu (UUR)*), two serine-tRNA genes (*tRNASer (UCN)* and *tRNASer (AGY)*), and two glutamate-tRNA genes (*tRNAGlu (NNN)* and *tRNAGlu (UUC)*), were present in the mitogenome. As already noted, the mitogenome of *B. bubo* comprised two CRs (CR1 and CR2). CR1 was 1581 bp long and was located between *NNN* and *tRNAPro*. CR2 was 1729 long and was located between *UUC* and *tRNAPhe*. Duplicate CR copies were also observed, and moderate sequence similarity (75–94%) was observed between them (Meng et al. 2020). Compared with the previously published mitogenome of Chinese *B. bubo* (MG681083), which is 18,952 bp long, a sequence similarity of 99.8% was observed in regions other than the D-loop (96.7%) and the pseudo-CR (99.7%).

**Figure 3.**
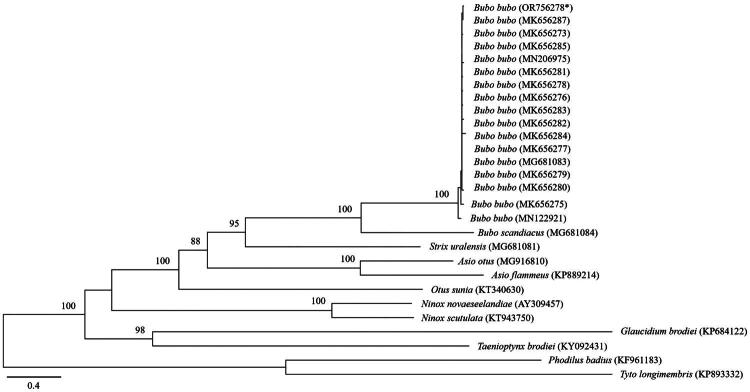
The phylogenetic tree was constructed based on the complete mitogenome of Korean *B. bubo* (OR756278) and 27 other sequences, using the maximum-likelihood (ML) methods, inferred from 1000 replicates. *Phodilus badius* (KF961183) and *Tyto longimembris* (KP893332) were set as out-groups. The nucleotide sequence matrix included 12 protein-coding genes. Bootstrap value above 50% in the ML analysis is indicated at each node. The mitochondrial genome sequenced in this study is shown with an asterisk. The accession number of the species in the tree: *Asio flammeus* (KP889214; Zhang et al. 2016), *Asio otus* (MG916810; Lee et al. 2018), *Bubo bubo* (OR756278; this study), *Bubo bubo* (MG681083; Kang et al. 2018), *Bubo bubo* (MN122921; A. Margaryan, unpublished data), *Bubo bubo* (MK656273–MK656287; Meng et al. 2020), *Bubo bubo* (MN206975; Z. Ren, unpublished data), *Bubo scandiacus* (MG681084; Kang et al. 2018), *Glaucidium cuculoides* (KY092431; Liu et al. 2019), *Ninox novaeseelandiae* (AY309457; Harrison et al. 2004), *Ninox scutulata* (KT943750; C.E. Park et al. unpublished data), *Otus sunia* (KT340630; Park et al. 2019), *Phodilus badius* (KF961183; Mahmood et al. 2014), *Strix uralensis* (MG681081; Kang et al. 2018), and *Taenioptynx brodiei* (KP684122; Sun et al. 2016).

Our phylogenetic analysis revealed that *B. bubo* was well placed within the family Strigidae (Figure 3), forming a sister clade with the Chinese *B. bubo*. Our dataset revealed that *B. bubo* was more closely related to the genus *Strix* than to any other genus. The mitogenome of Danish *B. bubo* (MN122921) barely varied from those of South Korean and Chinese *B. bubo*, as evidenced by the nearly identical sequences across these geographically distinct populations. This indicated a high genetic-conservation level within the species, possibly, highlighting recent gene flow among populations or slow mitogenome evolutions among this species. Such homogeneity across distant regions underscores the stability of the mitogenome of *B. bubo* despite their geographic distributions.

## Discussion and conclusions

The complete mitogenome sequence of *B. bubo* presented in this study offers significant insights into the genetic compositions of this bird and the evolutionary relationship of the species with other members in the genus *Bubo*. Our phylogenetic analysis confirmed that *Bubo* is more closely related to *Strix* than to any other genus considered in this study of owl systematics (Salter et al. 2020).

Further, the placement of *B. bubo* within the tribe Strigidae is consistent with the findings of the extant studies on owl taxonomy. Our analysis also revealed the existence of a close relationship between the Korean and Chinese *B. bubo* populations, indicating possible historical connections and gene flows between these adjacent populations. Furthermore, our findings underscore the importance of considering the regional genetic diversity and population dynamics in conservation efforts for this species. We present comprehensive molecular data on the mitogenome of the Korean *B. bubo* and provide valuable insights into the evolutionary relationships within the genus *Bubo*. This information will contribute to future conservation strategies and further studies on this avian species.

## Supplementary Material

Supplemental material.docx

## Data Availability

The genome sequence data that support the findings of this study are openly available in GenBank of NCBI at https://www.ncbi.nlm.nih.gov under the accession no. OR756278. The associated BioProject, SRA, and Bio-Sample numbers are PRJNA1139987, SRR29979503, and SAMN42801729, respectively.
