# Supplementary material for: Complete mitochondrial genome sequence of the South Korean Eurasian eagle-owl, Bubo bubo spp. kiautschenis (Strigiformes; Strigidae)
Source: Mitochondrial DNA B Resour. 2025 Nov 9;10(12):1112–5. doi: 10.1080/23802359.2025.2528345 (PMC12599151; doi:10.1080/23802359.2025.2528345)

**Supplemental material 1.** Mapping statistics of the mitochondrial sequence data of *Bubo bubo* Bbu-01 to reference genome (GenBank accession number, MG681083) and Mapping graph of the mitochondrial sequence data of *Bubo bubo* Bbu-01 to reference.

| Reference genome | Start base position | End base position | No. of reads | No. of reference base mapped | Coverage | Minimum sequencing depth | Mean sequencing depth | Max sequencing depth |
| --- | --- | --- | --- | --- | --- | --- | --- | --- |
| MG681083 | 1 | 18952 | 1,093,073 | 18952 | 100% | 998 | 6,957 | 23,470 |


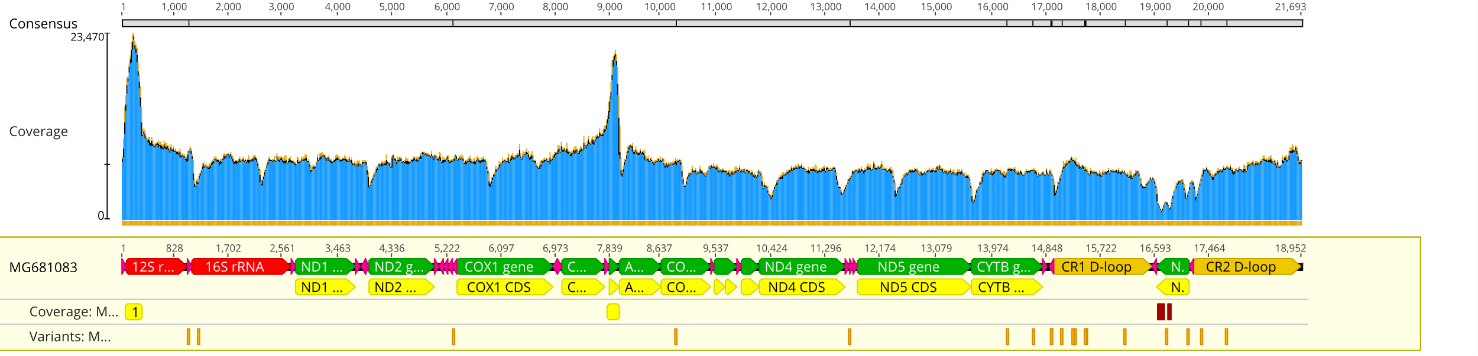

Supplement: Supplemental material.docx [file TMDN_A_2528345_SM7965.docx]
